# Medical Legislation in Tennessee

**Published:** 1875-09

**Authors:** 


MEDICAL LEGISLATION IN TENNESSEE.—At a recent meeting of the Knox County Medical Society, a committee which was appointed to make suggestions regarding a bill for the regulation of the practice of medicine, pending before the Legislature of Tennessee, offered the following:

"That the less the medical profession, as a profession, has to do in identifying itself with legislative enactments, and the more its members are left each to be his own cicerone before the public, and answerable to public opinion and the laws as they are for any damage he may commit, the more respect will be given to the whole profession."

The following resolution, after considerable discussion, was also adopted:

"*Resolved,* That the Secretary be instructed to request Senator Ellis to place a clause in his bill, making it a misdemeanor for any one to claim to have graduated in a Medical College, or taken the degree of M.D., when such is not the case."

This is an evidence of the growth of medical sentiment in regard to special legislation, which is certainly very encouraging. *Medical Record.*

